# Diagnostic and Prognostic Validity of Proadrenomedullin among Neonates with Sepsis in Tertiary Care Hospitals of Southern India

**DOI:** 10.1155/2018/7908148

**Published:** 2018-08-01

**Authors:** Raja Kannan, Suchetha S. Rao, Prasanna Mithra, B. Dhanashree, Shantharam Baliga, Kamalakshi G. Bhat

**Affiliations:** ^1^Senior Resident, Department of Pediatrics, Kasturba Medical College Mangalore, Manipal Academy of Higher Education, Mangalore, Karnataka 575001, India; ^2^Associate Professor, Department of Pediatrics, Kasturba Medical College Mangalore, Manipal Academy of Higher Education, Mangalore, Karnataka 575001, India; ^3^Associate Professor, Department of Community Medicine, Kasturba Medical College Mangalore, Manipal Academy of Higher Education, Mangalore, Karnataka 575001, India; ^4^Associate Professor, Department of Microbiology, Kasturba Medical College Mangalore, Manipal Academy of Higher Education, Mangalore, Karnataka 575001, India; ^5^Professor, Department of Pediatrics, Kasturba Medical College Mangalore, Manipal Academy of Higher Education, Mangalore, Karnataka 575001, India; ^6^Professor and Head, Department of Pediatrics, Kasturba Medical College Mangalore, Manipal Academy of Higher Education, Mangalore, Karnataka 575001, India

## Abstract

**Introduction:**

To evaluate Proadrenomedullin (Pro-ADM) as the diagnostic and prognostic marker in neonatal sepsis.

**Materials and Methods:**

In this cross-sectional study, Pro-ADM levels were estimated in 54 neonates with clinical sepsis and positive sepsis screen (cases) and 54 controls without clinical sepsis. Repeat Pro-ADM levels were estimated after 72 hours in cases. Pro-ADM levels were compared with the clinical outcome.

**Results and Discussion:**

Median Pro-ADM levels in cases were 31.8 (IQR: 27.8-39.4) pmol/ml which was significantly higher than controls 5.1 (IQR; 3.1-7.7) pmol/ml. From the constructed ROC curve, a value of 14.5 pmol/ml was taken as the cut-off for sepsis. Pro-ADM had 100% sensitivity, specificity, and positive predictive values (PPV) in detecting sepsis at 14.5 pmol/ml. Among cases, a decrease in Pro-ADM level by 10 pmol/ml was associated with 99% survival. Pro-ADM value of 35 pmol/ml had 100% specificity and PPV in predicting mortality.

**Conclusion:**

Pro-ADM can be used as a single biomarker for detecting neonatal sepsis, predicting clinical outcome and prognosis.

## 1. Introduction

Sepsis is the leading cause of neonatal morbidity and mortality. World Health Organization (WHO) estimates that, out of 4 million neonatal deaths around the world every year, over 35% are due to sepsis [1]. Globally, neonatal mortality is on decreasing trend [2]. Over the last two decades, there is a 47% reduction in Neonatal Mortality Rate (NMR) from 36 deaths to 19 deaths per 1000 live births. However neonatal mortality still constitutes a considerable proportion of under-five deaths [2, 3].

Early identification of sepsis is challenging because neonates present with nonspecific symptoms and signs [4]. This emphasizes the need to detect sepsis by laboratory investigations at the earliest for prompt initiation of treatment.

The routine sepsis screen like Total Leucocyte Count (TLC), Immature to Total neutrophil ratio (IT Ratio), Micro Erythrocyte Sedimentation Rate (Micro ESR), and C-reactive protein (CRP) is not specific indices of sepsis [4–6]. This leads to the search for a new biochemical marker which helps in diagnosis of sepsis early.

Adrenomedullin (ADM) is a potent vasodilating peptide which is widely expressed and synthesized during sepsis. However, accurate measurement of ADM is technically challenging because it has a short half-life (t1/2–22 min) and hence gets cleared rapidly from circulation. Proadrenomedullin (Pro-ADM) is the most stable component of ADM. Measurement of Pro-ADM is an indirect measurement of ADM [7–10].

There are limited studies in this regard. Hence we carried out this study to assess the significance of Pro-ADM as a diagnostic and prognostic marker in neonatal sepsis.

## 2. Materials and Methods

This hospital-based case control study was carried out in the Neonatal Intensive Care Unit (NICU) of tertiary care hospitals attached to Kasturba Medical College (Manipal Academy of Higher Education), Mangaluru in Coastal South India, between November 2016 and April 2017. In this study, neonates with clinical sepsis and positive sepsis screen [11] were included in sepsis group (cases) and neonates without clinical sepsis as the control group. After obtaining clearance from Institutional Ethics Committee (IEC), necessary permissions were taken from the hospital authorities. The study hospitals were visited for data collection.

Clinical sepsis was defined as neonates with 2 or more of the following features which includes (1) temperature instability, (2) cardiovascular instability, (3) respiratory instability, (4) gastrointestinal instability, (5) petechial rash or sclerema, and (6) nonspecific features [10]. Neonates with cardiac failure, severe intracranial bleeding (Grade 3 or 4), persistent pulmonary hypertension of newborn, renal failure, preterm < 32 weeks of gestation, surgical conditions, previously exposed to antibiotics, and maternal preeclampsia were excluded from the study. Sepsis screening was considered positive if two or more of the following parameters were observed which includes TLC <4000 x10^9^/L or >20000 x 10^9^/L, IT Ratio > 0.2, Micro ESR > 15 mmHg, and CRP > 6 mg/dl [11].

The sample size was taken as 54 in each group based on sample size nomogram for estimating sensitivity and specificity of a medical test [12].

Venous blood sample for culture and Pro-ADM (Pro-ADM1) was collected from 54 neonates in sepsis group. Venous blood sample for Pro-ADM (Pro-ADM1) estimation was collected from neonates in the control group. Pro-ADM (Pro-ADM1) level was measured by Pro-ADM Enzyme Linked Immuno Sorbent Assay (ELISA) kit (Wuhan Fine Biological Technology Co. Ltd.) which is a sandwich ELISA. The Pro-ADM1 levels of sepsis group were compared with those of controls. From the neonates with sepsis, venous blood sampling was repeated after 72 hours for estimation of Pro-ADM (Pro-ADM2). In neonates with sepsis, initial Pro-ADM (Pro-ADM 1) levels were compared with the repeat levels (Pro-ADM2) to know the outcome of sepsis.

The collected data were coded and entered into Statistical Package for Social Sciences (SPSS) version 11.5. Results were expressed as proportion and median scores with interquartile range (IQR). The role of Pro-ADM for diagnosis of neonatal sepsis and outcome was determined by Receiver Operating Characteristic curve (ROC) and calculating area under the curve. The ROC curve was used to predict the optimum cut-off values. Sensitivity and specificity of Pro-ADM were calculated. Prognostic significance was calculated using chi-square test. For comparison across the group, Mann–Whitney U test and chi-square test were used. A p value <0.05 was considered as statistically significant.

## 3. Results

Table 1 depicts the comparison of demographic data and Pro-ADM levels in the study population. The median Pro-ADM1 level in control group was 5.1 (IQR: 3.1-7.7) pmol/ml and in sepsis group was 31.8 (IQR: 27.8-39.4) pmol/ml. This difference was statistically significant.

**Figure 1:**
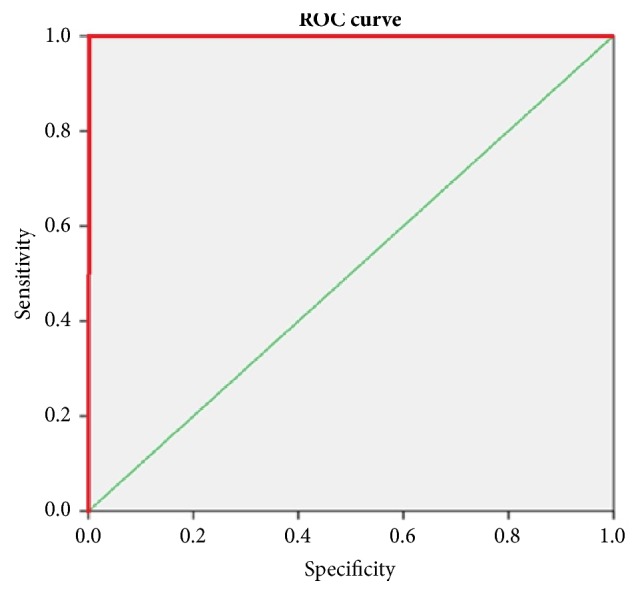
ROC curve for Pro-ADM1 in predicting sepsis in the study population.

ROC curve was generated (Figure 1) for defining the value for sepsis using Pro-ADM1 level compared with the presence or absence of sepsis. Based on the points of coordinates value of 14.5 pmol/ml was taken as a cut-off to detect neonatal sepsis. In control group, all neonates had normal Pro-ADM1 values (<14.5 pmol/ml), and in sepsis group, all neonates had high Pro-ADM1 values (>14.5 pmol/ml) (Table 2). The Area Under Curve (AUC) was 1. At a value of 14.5 pmol/ml, Pro-ADM1 had a sensitivity and specificity of 100%, the positive predictive value of 100%, and negative predictive value of 100% in detecting sepsis.

Pro-ADM2 was measured in sepsis group after 72 hours. During the study period, four neonates expired within 72 hours of measurement of Pro-ADM1. Hence Pro-ADM2 was measured only in 50 septic neonates. The values of Pro-ADM1 and Pro-ADM2 were compared with the outcome of the sepsis. Of the total 50 cases, Pro-ADM2 levels were decreased in 80% of neonates and increased in 20% of neonates on subsequent measurements. Out of the 40 cases with decreased Pro-ADM2 levels, 92.5% neonates improved and 7.5% neonates expired. Among 10 cases with increased Pro-ADM2 levels, 90% of neonates expired and 10% neonates survived. This result had a P value <0.001 which was statistically significant (Table 3).

**Figure 2:**
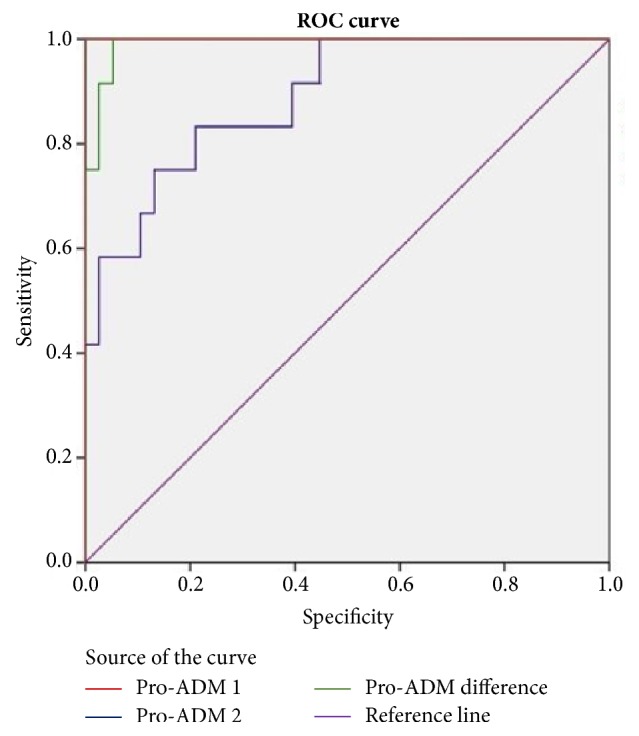
ROC curve for Pro-ADM in predicting the survival in the study population.

ROC curve was generated based on the difference in the Pro-ADM1 and Pro-ADM2 compared with the outcome. AUC was 0.991. Based on the points of coordinates, it was observed that decrease in Pro-ADM2 level by 10 pmol/ml is associated with 99% survival of neonates (Figure 2). This result was statistically significant (P value <0.001).

ROC curve was generated to find out the level of Pro-ADM1 among sepsis group which can predict the chances of mortality (Figure 2). Based on the points of coordinates, giving optimum levels of sensitivity and specificity regarding the survival of neonates, a level of 35 pmol/ml was taken as a cut-off to predict mortality.

Of the total 54 septic neonates, 38 (70.4%) improved and 16 (29.6%) expired. Among the improved neonates, 81.6% had Pro-ADM1 levels less than 35 pmol/ml, and 19.4% had Pro-ADM1 levels more than 35 pmol/ml. Of the 16 expired neonates, 87.5% had a Pro-ADM1 level more than 35 pmol/ml, and 12.5% had a Pro-ADM1 level less than 35 pmol/ml (Table 4). This analysis was statistically significant (P value <0.001).

At a cut-off 35 pmol/ml, Pro-ADM1 had a sensitivity of 88%, specificity of 82%, the positive predictive value of 67%, and the negative predictive value of 88% to predict mortality in septic neonates.

Of the 54 septic neonates, 16 (29.6%) neonates had growth in blood culture. Median Pro-ADM levels in blood culture positive neonates in our study were 36.2 (IQR: 29.5-39.8) pmol/ml which was significantly higher than control group 5.1 (IQR: 3.1-7.7) pmol/ml. The mortality rate in culture positive sepsis was 37.5%. Neonates who survived in culture positive sepsis had a drop of >10 pmol/ml on subsequent measurement.

## 4. Discussion

Sepsis is a significant cause of mortality and morbidity in the neonatal period. It is a challenge to detect sepsis because of its nonspecific clinical presentation. Hence, laboratory testing is mandatory to identify sepsis. Most of the routinely used investigations like TLC, IT Ratio, Micro ESR, and CRP are not specific indices of sepsis. Hence there is a need for a biomarker with high sensitivity, specificity, and predictive values which can be used solely to detect sepsis.

Pro-ADM as explained by Christ Crain et al. and Adamty et al. was elevated in sepsis due to various reasons, the primary reason being an excessive synthesis of Pro-ADM during sepsis. This is because ADM gene is widely expressed during sepsis. The other reason is decreased clearance of Pro-ADM by kidney and lung. They also explained that decreased systemic vascular resistance observed during sepsis could also be the reason for elevated levels of Pro-ADM [13, 14].

In the present study, the median Pro-ADM level was higher in septic neonates 31.8 (IQR: 27.8-39.4) pmol/ml compared to controls 5.1 (3.1-7.7) pmol/ml. A similar finding was observed by Ashraf et al., where the mean Pro-ADM in cases was 4.7±3.2 pmol/ml and in controls was 0.79±0.31 pmol/ml. Earlier studies by Hagag et al. and Yun Cao et al. have also observed higher Pro-ADM levels in sepsis group compared to the control group [15, 16].

In a study conducted by Mehmet et al., Pro-ADM levels were higher in proven sepsis (53.4 ± 34.4 pmol/ml) group than clinical sepsis (18.2 ± 27.1 pmol/ml) group. A similar significant difference of Pro-ADM between culture-proven sepsis 2.49 ± 1.29 pmol/ml and clinical sepsis 1.90 ± 1.12 pmol/ml was demonstrated by Yun Cao et al. [16, 17]. However, in the present study, 29.6% of cases had culture-proven sepsis. The median Pro-ADM level in our blood culture positive neonates was 36.2 (IQR: 29.5-39.8) pmol/ml which was significantly higher than control group 5.1(IQR: 3.1-7.7) pmol/ml.

In an earlier study, at the cut-off value of 3.9 pmol/ml, the sensitivity of Pro-ADM in detecting neonatal sepsis was 91.6%, its specificity was 87.4%, its positive predictive value was 91.3%, and its negative predictive value was 90.4% [18]. At the same value in the present study, the sensitivity of Pro-ADM in detecting sepsis was 100%, specificity was 37.1%, the positive predictive value was 61.4%, and the negative predictive value was 100%.

At the cut-off level of 14.5 pmol/ml for detection of sepsis in the present study, all controls had normal Pro-ADM values, and all cases had high Pro-ADM values. Pro-ADM at this value had 100% sensitivity, specificity, and predictive values in detecting sepsis.

In our study, apart from knowing the diagnostic significance of Pro-ADM in sepsis, an attempt was made to find out the prognostic significance also. This was made possible by serial estimation of Pro-ADM in cases after 72 hours and comparing the difference in the outcome of neonates.

It was observed that 90% of neonates with increased Pro-ADM2 expired and 92.5% of neonates with decreased Pro-ADM2 survived. It was also seen that decrease in Pro-ADM2 level by 10 pmol/ml is associated with 99% survival. It is thus evident that serial monitoring of Pro-ADM levels had a significant role in predicting prognosis. To the best of our knowledge, serial monitoring of Pro-ADM and its utility in predicting sepsis has not been reported so far in the literature.

We also observed that the median Pro-ADM level in the expired group was 43.2 (IQR: 29.4-48.1) pmol/ml which was significantly higher than the median Pro-ADM level seen in survived group 28.4 (IQR: 17.9-41.4) pmol/ml. A similar finding was reported by earlier studies as well [15, 18]. It was observed that Pro-ADM at a cut-off 35 pmol/ml can significantly predict the chances of mortality and is associated with 67% mortality.

## 5. Conclusion

To conclude, the results of the study strongly suggest the use of Pro-ADM as a single biomarker for early diagnosis. Serial measurement of Pro-ADM can help in predicting the outcome in neonatal sepsis. Hence Pro-ADM is a promising prognostic marker in neonatal sepsis. Further studies are required involving multiple centers to establish a specific Pro-ADM level for diagnosis and predicting clinical outcome.

## Figures and Tables

**Table 1:** Demographic data and Pro-ADM1 levels in the study population.

**Characteristics**	**Control group (n=54)** No. (%)	**Sepsis group (n=54)** No. (%)
**Gestational Age**		
Term	31 (57.4)	29 (53.7)
Preterm	23 (42.6)	25 (46.3)
**Gender**		
Male	24 (44.4)	28 (51.9)
Female	30 (55.6)	26 (48.1)
**Place Of Birth**		
Inborn	54 (100)	52 (96.3)
Outborn	00 (0)	02 (03.7)
**Mode Of Delivery**		
Vaginal Delivery	34 (63)	39 (72.2)
Elective LSCS	10 (18.5)	02 (03.7)
Emergency LSCS	10 (18.5)	13 (24.1)
	**Median**	Median
**Birth Weight**	2.55 (IQR:2.3-2.88) kg	2.28 (IQR:1.8-2.47)kg
**Pro-ADM**	5.1 (IQR:3.1-7.7) pmol/ml	31.8 (IQR:27.8-39.4) pmol/ml.

**Table 2:** Comparison of Pro-ADM1 among sepsis group and control with the cut-off level of 14.5pmol/ml.

	**Control group** **(n=54)** **No. (%)**	**Sepsis group** **(n=54)** **No. (%)**	**P value**
**Normal Pro-ADM1** **< 14.5pmol/ml**	54 (100)	00 (0)	<0.001^*∗*^
**High Pro-ADM1** **> 14.5pmol/ml**	00 (0)	54 (100)	

^**∗**^P  value significant at 0.05 level.

**Table 3:** Comparison of change in levels of Pro-ADM1 and Pro-ADM2 and outcomes in the sepsis group.

	**Improved (n=38)** **No. (%)**	**Expired (n=12)** **No. (%)**	**P value**
**Pro-ADM2 Increased**	01 (2.6)	09 (75)	<0.001^*∗*^
**Pro-ADM2 Decreased**	37 (97.4)	03 (25)	

^*∗*^P  value significant at 0.05 level.

**Table 4:** Comparison of Pro-ADM1 with cut-off 35pmol/ml and outcome in the study population.

	**Improved (n=38)** **No. (%)**	**Expired (n=16)** **No. (%)**	**P value**
**Pro-ADM1** **<35pmol/ml**	31 (81.6)	02 (12.5)	<0.001^*∗*^
**Pro- ADM1** **>35pmol/ml**	07(19.4)	14 (87.5)	

^*∗*^P value significant at 0.05 level.

## Data Availability

The data used to support the findings of this study are available from the corresponding author upon request.

## Supplementary Materials

Supplementary Materials Datasets used and analyzed during the current study.
